# Directed endozoochorous dispersal by scavengers facilitate sexual reproduction in otherwise clonal plants at cadaver sites

**DOI:** 10.1002/ece3.8503

**Published:** 2022-01-26

**Authors:** Mie Prik Arnberg, Shane C. Frank, Rakel Blaalid, Marie Louise Davey, Amy Elizabeth Eycott, Sam M. J. G. Steyaert

**Affiliations:** ^1^ Faculty of Biosciences and Aquaculture Nord University Steinkjer Norway; ^2^ University of South‐Eastern Norway Bø Norway; ^3^ Department of Natural History University Museum of Bergen Bergen Norway; ^4^ Norwegian Institute for Nature Research Trondheim Norway

**Keywords:** cadaver decomposition island, directed dispersal, Ericaceae, recruitment window of opportunity, reproductive paradox

## Abstract

1. The regeneration niche of many plant species involves spatially and temporally unpredictable disturbances, called recruitment windows of opportunity. However, even species with clear dispersal adaptations such as fleshy berries may not successfully reach such elusive regeneration microsites. Ericaceous, berry‐producing species in the northern hemisphere demonstrate this dispersal limitation. They are said to display a reproductive paradox owing to their lack of regeneration in apparently suitable microsites despite considerable investment in producing large quantities of berries.
2. Cadavers generate vegetation‐denuded and nutrient‐rich disturbances termed cadaver decomposition islands (CDIs). Cadavers attract facultative scavengers with considerable capacity for endozoochorous seed dispersal. We hypothesize that CDIs facilitate recruitment in berry‐producing ericaceous species due to endozoochorous dispersal directed toward favorable microsites with low competition.
3. We examined seedling establishment within a permanent, semi‐regular 10 × 10 m grid across an ungulate mass die‐off on the Hardangervidda plateau in southeastern Norway. Competing models regarding the relative importance of factors governing recruitment were evaluated, specifically cadaver location (elevated seed rain) and microsite conditions (competition).
4. We found that CDIs did facilitate seedling establishment, as cadaver density was the best predictor of seedling distribution. Other important factors governing seedling establishment such as percentage cover of soil and vascular plants alone were inadequate to explain seedling establishment.
5. *Synthesis*: This study provides a novel understanding of sexual reproduction in species with cryptic generative reproduction. The directed nature of endozoochorous dispersal combined with long‐distance dispersal abilities of medium to large vertebrate scavengers toward cadavers allows plants to exploit the advantageous but ephemeral resource provided by CDIs.

## Graphical abstract

We disentangle the reproductive paradox of ericaceous berry‐producing species and demonstrate a pathway of successful sexual reproduction in these otherwise clonal plants. Berry‐consuming vertebrate scavengers provide directed endozoochorous dispersal toward cadavers, resulting in higher probability of seedling establishment at cadaver decomposition islands (CDIs). This mechanism allows plants to exploit the advantageous but ephemeral microsite provided by CDIs.

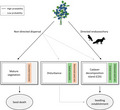

## 1 INTRODUCTION

Berry‐producing ericaceous shrubs are keystone species throughout many of the temperate, boreal and alpine habitats in the Northern hemisphere, with important effects on ecosystem function (Mallik, 2003). Ericaceous vegetation dominates the boreal understory, regulating seedling survival and subsequently species composition (Nilsson & Wardle, 2005). Removal of this functional group has detrimental effects on soil microbial activity and soil quality (Fanin et al., 2019). Furthermore, their vegetative structures and berries are an important food source for many mammal, bird, and insect species (Atlegrim, 1989; Dahlgren et al., 2007; Wegge & Kastdalen, 2008).

While their importance for ecosystem function is well known, berry‐producing genera such as *Vaccinium* and *Empetrum* present a reproductive paradox (Kloet & Hill, 1994). These species invest in the annual production of large crops of conspicuous fleshy fruits (berries) that contain viable seeds. The berries are consumed by a wide range of mammals and birds (e.g., brown bear, *Ursus arctos*; red fox, *Vulpes vulpes*; tetraonids and corvids) and are an adaptation for endozoochory, that is, seed dispersal via ingestion by animals (Howe & Smallwood, 1982; Willson, 1993). Despite their substantial investment in sexual reproduction, *Vaccinium* and *Empetrum* spp. are reported to propagate almost exclusively clonally (Hautala et al., 2001; Welch et al., 2000). Furthermore, their seeds are consistently under‐represented in the soil seed bank and seedlings are rarely observed in the wild (Hester et al., 1991; Kloet & Hill, 1994; Ranwala & Naylor, 2004; Welch et al., 2000). This apparent reproductive paradox and its implications for the population biology of many ericaceous berry‐producing shrubs are poorly understood and infrequently addressed (Kloet & Hill, 1994; Welch et al., 2000).

Recruitment in plants via endozoochory is closely tied to mutualistic interactions with frugivorous and omnivorous animal partners (Schupp et al., 2017). Animals remove fruits for resource acquisition and simultaneously provide dispersal services for plants by transporting and depositing seeds. This allows plant offspring to escape higher density‐ and distance‐dependent mortality near conspecifics (Janzen–Connell mechanism; Comita et al., 2014), to locate ephemeral microsites suited for establishment and even to colonize newly available habitat patches (Escribano‐Avila et al., 2014; Howe & Smallwood, 1982). The seed dispersal effectiveness, that is, contribution of a dispersal agent to the recruitment likelihood of a given plant species, is the outcome of a complex and multistage process (Schupp et al., 2010). Firstly, fruits must be detected and ingested. The number of seeds dispersed is dependent on the number of seeds removed per visit and the number of visits, which is related to the abundance of dispersers and their feeding behavior. Secondly, the recruitment probability of dispersed seeds will be affected by the rate of successful scarification versus the rate of viability loss incurred during passage through the gastro‐intestinal system and the quality of the seed deposition site (Schupp et al., 2010 and Schupp et al., 2017).

The microsite of seed deposition is critical for seed dispersal effectiveness (Schupp et al., 2010), as it must accommodate species‐specific requirements for germination and subsequent seeding establishment and survival (Eriksson & Ehrlén, 1992; Grubb, 1977). Both abiotic and biotic microsite conditions (e.g., competition, nutrients, and seed predation) will be spatially and temporarily variable throughout a landscape (Riedel et al., 2005; Spasojevic et al., 2013). Situations where favorable microsite conditions are relatively short‐lived are referred to as recruitment “windows of opportunity” (RWOs; Jelinski & Cheliak, 1992). Seed addition experiments have demonstrated that ericaceous species readily germinate and establish in suitable RWO‐type microsites (Eriksson & Fröborg, 1996; Manninen & Tolvanen, 2017). These RWOs consist of disturbances within mature stands of ericaceous vegetation that remove potential barriers to recruitment such as field‐ and ground‐layer vegetation (i.e., competition) and are typically characterized by high moisture and organic soil content (Eriksson & Fröborg, 1996; Graae et al., 2011). Such RWOs, however, are often spatio‐temporally unpredictable, highlighting the need for dispersing seeds to reach suitable areas for germination, seedling survival, and growth (Eriksson & Fröborg, 1996; Hautala et al., 2001; Manninen & Tolvanen, 2017).

Cadavers of large animals generate small‐scale disturbances in the landscape and may represent an ecologically important RWO for berry‐producing ericaceous species (Steyaert et al., 2018). Nearby and underneath a cadaver, vegetation is either killed by abrupt shifts in soil nutrients and acidity or smothered, creating vegetation‐denuded and nutrient‐rich patches, termed “cadaver decomposition islands” (CDIs; Carter et al., 2007; Towne, 2000). In addition, cadavers are a food source for several scavenging species during decomposition (DeVault et al., 2003) leading to an aggregation of fecal deposition (Steyaert et al., 2018) that further contributes to organic input at the CDI (Carter et al., 2007). Thus, the disturbance from a CDI increases nutrient availability and removes competition in spatially discrete areas (Barton et al., 2016; Bump et al., 2009) which closely resemble the microsites described as ideal for ericaceous seedling establishment.

The majority of scavenging vertebrates that utilize cadavers as a food source are facultative scavengers (DeVault et al., 2003), that is, they are not solely dependent on carrion material and can include plant material as part of their diet. For example, during peak fruiting season, berries can constitute up to 30% of the diet in red fox (Needham et al., 2014) and as much as 82% in brown bears (Dahle et al., 1998). Such facultative scavengers often have large home ranges and thus considerable capacity as vectors for long‐distance endozoochorous seed dispersal (González‐Varo et al., 2013; Schaumann & Heinken, 2002). In addition, the attractiveness of cadavers for berry‐dispersing vertebrate scavengers suggests that such long‐distance seed dispersal may be directed toward the CDIs (Steyaert et al., 2018).

The directed dispersal hypothesis, namely the disproportionate arrival of seeds at targeted microsites with particularly favorable conditions for recruitment, has been proposed to explain some plants’ costly investment in nutritious fruit production (Howe & Smallwood, 1982; Wenny, 2001). Directed endozoochorous seed dispersal toward CDIs by facultative scavengers has been postulated as an important mechanism for sexual reproduction in ericaceous shrub species. Steyaert et al. (2018) demonstrated that ungulate cadavers are endpoints for directed endozoochory at a wild tundra reindeer (*Rangifer tarandus*) mass mortality event in southcentral Norway. They found that defecation by scavengers was concentrated around cadavers and scavenger feces contained large numbers of viable seeds of the berry‐producing ericaceous shrub crowberry (*Empetrum nigrum*). However, seed dispersal is only effective if it results in seedling establishment (Schupp et al., 2010, 2017).

**FIGURE 1:**
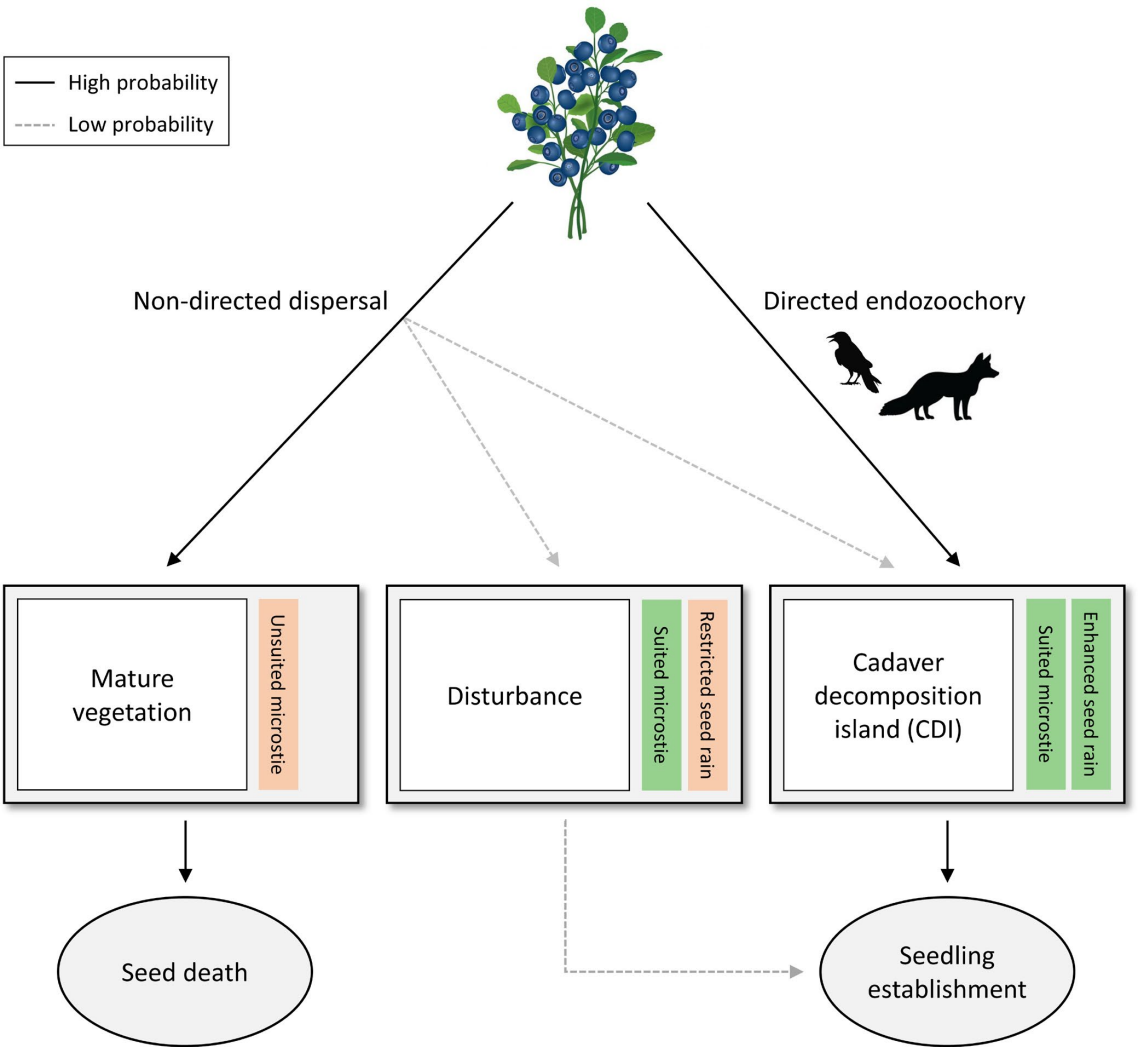
Schematic diagram showing directed seed dispersal by facultative scavengers toward cadaver decomposition islands as a pathway of successful sexual reproduction in berry‐producing ericaceous shrub species. Non‐directed dispersed seeds have a limited encounter rate with temporally and spatially unpredictable recruitment windows of opportunity. By linking the endozoochorous dispersal capacity of scavenging vertebrates and cadaver decomposition islands, seed limitation is overcome at ideal microsites due to (1) vectors facilitating their (2) directed dispersal (i.e., enhanced seed rain) into (3) favorable microsites for seedling establishment

We aim to further disentangle the reproductive paradox described for berry‐producing ericaceous shrubs by extending the finding of Steyaert et al. (2018) that cadavers aided in directed seed dispersal to incorporate the seedling establishment stage. We hypothesize that seedlings of berry‐producing ericaceous species are more likely to establish within CDIs due to (1) vectors facilitating their (2) directed dispersal into (3) favorable microsites (Figure 1). By examining seedling establishment in and around the same large CDI as Steyaert et al. (2018), we assess (I) whether cadaver presence facilitates successful seedling establishment and (II) the influence of other microsite conditions potentially important for seedling establishment such as reduced competition within the CDI. We predict that seedling occurrence will be more likely in close proximity to cadavers due to the combined effect of enhanced seed rain and favorable microsite conditions.

## 2 MATERIALS AND METHODS

### 2.1 Study site

The study was conducted at the site of an ungulate mass die‐off event near the Vesle Saure Lake (N: 60.021, Lon: 8.034) on the mountainous plateau of Hardangervidda in Southeastern Norway, previously described by Steyaert et al. (2018). The site is located 1220 m asl and is part of a large alpine tundra ecosystem. On August 26, 2016, a lightning strike killed almost an entire herd (*n* = 323) of wild tundra reindeer (*Rangifer tarandus*). National authorities removed the heads from all dead reindeer for disease monitoring, but the remaining biomass was left on site. The cadavers are distributed over a relatively small area (240 × 100 m), with the highest density confined to a 50 × 50 m area (Appendix 1).

**FIGURE 2:**
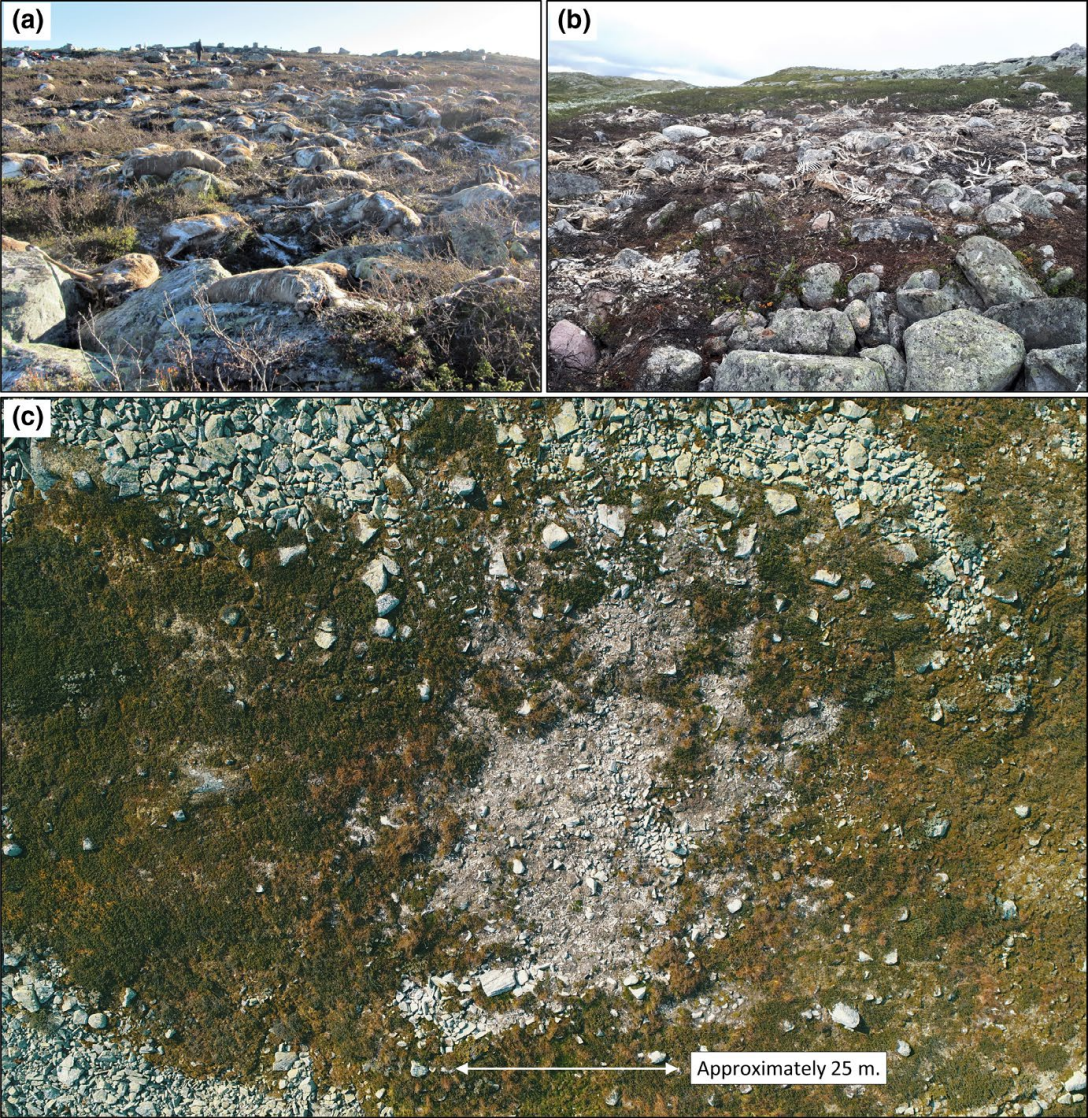
The 323 reindeer cadavers have transformed the vegetation community drastically at the mass die‐off site from 2016 (year of death) to 2019. (a) October 2016: The cadavers are relatively intact and the surrounding vegetation is alive. August 2019: (b) All soft tissue have either been removed by scavengers or decomposed while vegetation in the immediate vicinity has died off; (c) The mass die‐off site seen from 60‐m elevation. In the most cadaver‐intense area one large cadaver decomposition island (CDI) has formed, surrounded by several smaller CDIs

**FIGURE 3:**
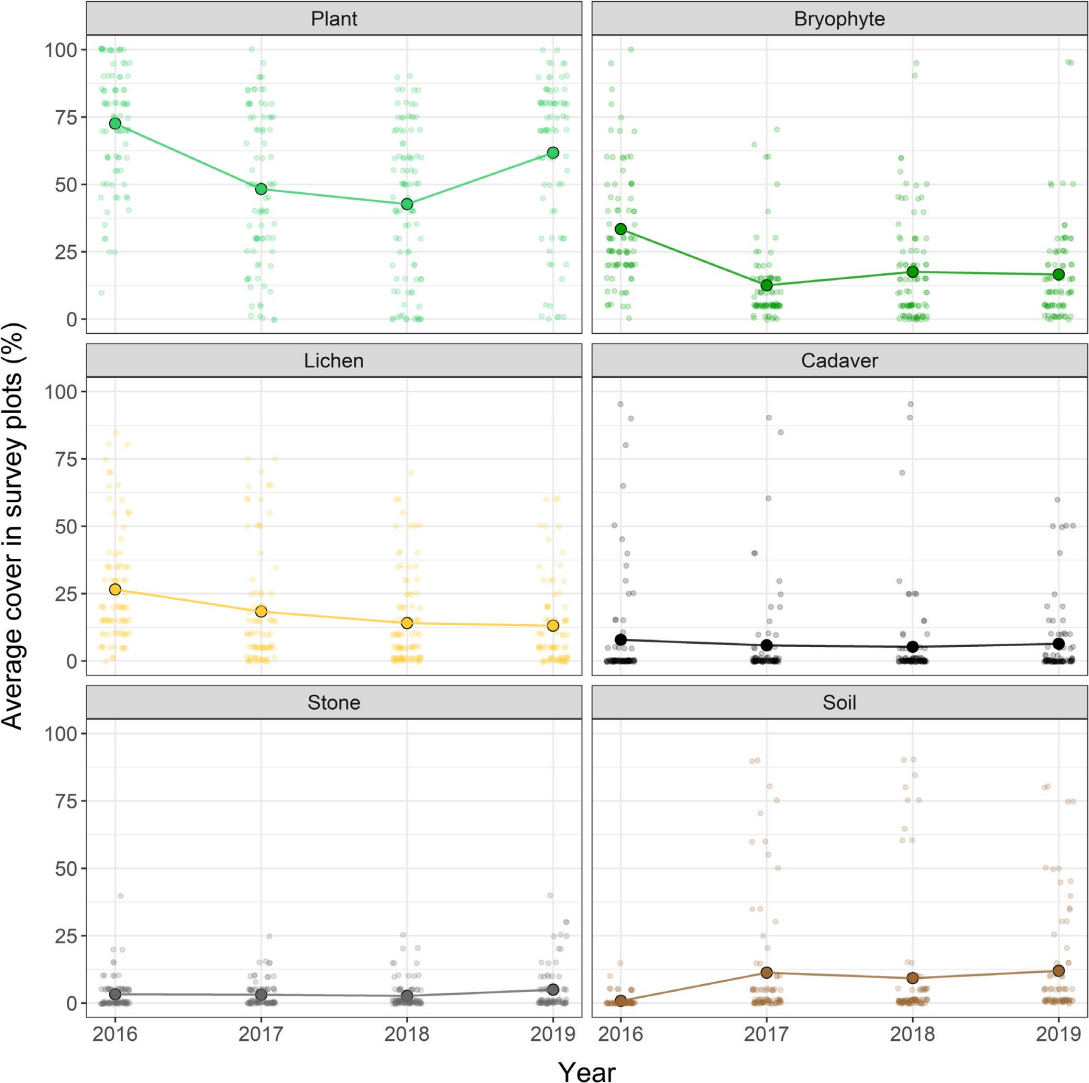
Average percentage cover of the functional groups (vascular plants, bryophytes, and lichen) and other microsite characteristics including persistent cadaver remains, stone, and soil within survey plots at the mass die‐off site from 2016 to 2019

The plant community is relatively species poor. In 2016, the field layer was dominated by dwarf birch (*Betula nana*) and ericaceous shrub species interspersed with graminoids and herbs and the ground layer had a consistent cover of bryophytes and lichens. However, over the course of 3 years the plant community within the most cadaver‐intense area had drastically changed. In 2019, the decomposing cadavers had created one large (25 × 25 m) and several small CDIs. Extensive areas were dominated by bare soil and re‐establishing bryophytes and graminoids (Figures 2 and 3).

Several vertebrate species, including scavengers, have been observed during previous fieldwork and camera trapping campaigns at the study site. Among those were corvids (common raven *Corvus corax* and hooded crow *C*. *cornix*), golden eagle (*Aquila chrysaetos*), foxes (red fox *Vulpes* and artic fox *V*. *lagopus*), wolverine (*Gulo gulo*), and several rodent species (e.g., in the Arvicolinae; Steyaert et al., 2018). Scavenging species continued to use the mass die‐off site after the initial mortality event in 2016. Scats showed that mesopredator and corvid occurrence was highly concentrated around the most cadaver‐dense part of the site in both 2017 and 2018 (Frank et al., 2020; Steyaert et al., 2018), although corvid scat density was considerably reduced in 2018 (Frank et al., 2020). Observations of scavengers and scats made during fieldwork coincided with the ericaceous berry‐ripening and dispersal season (August).

### 2.2 Data collection

**FIGURE 4:**
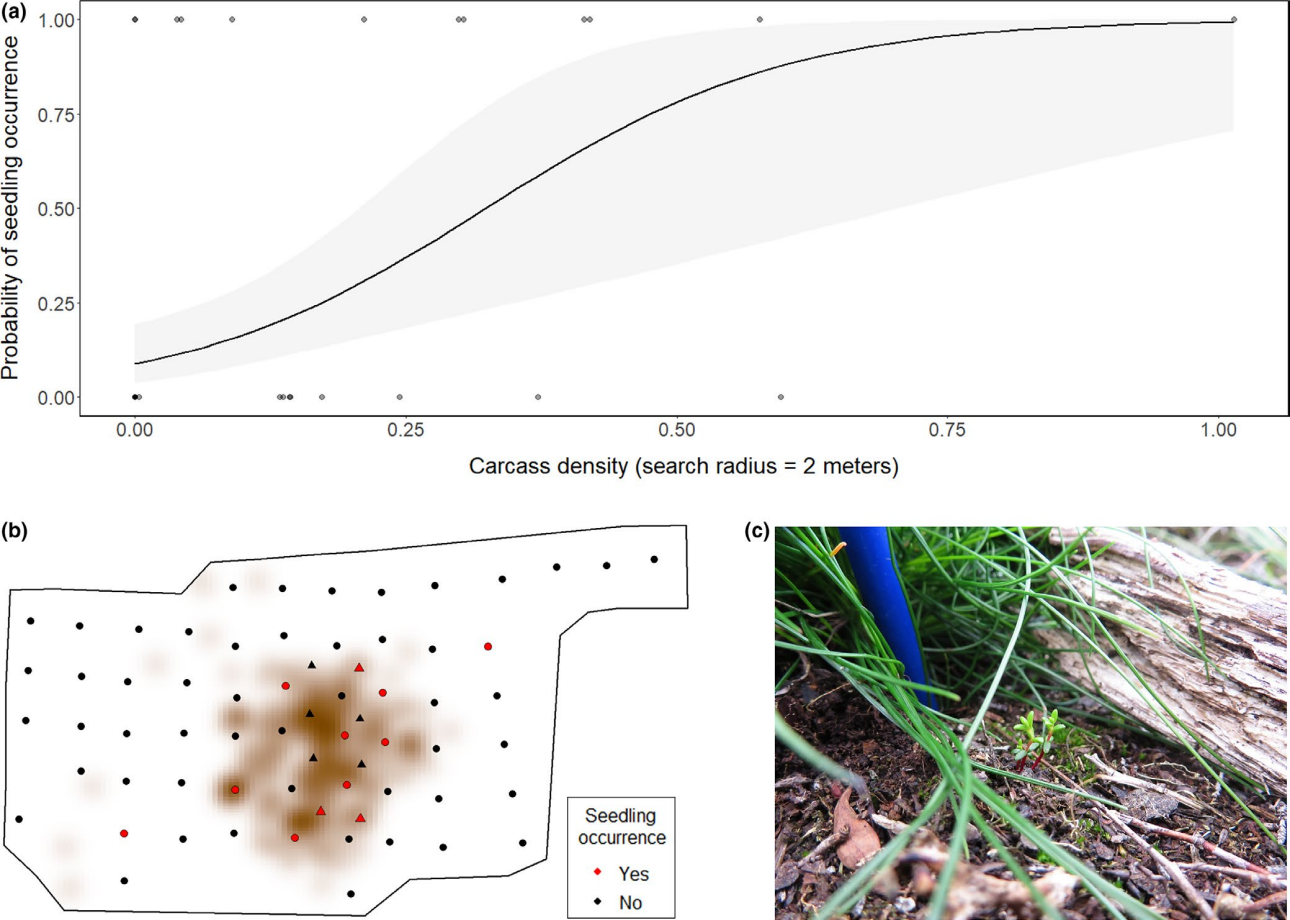
(a) The probability of seedling occurrence of berry‐producing ecricacuous species was positively correlated with cadaver density (search radius 2 m). The solid line is the Bernoulli generalized linear model predicted probability of seedling occurrence (0 = no seedling occurrence, 1 = seedling occurrence) relative to cadaver density. The 95% confidence interval for the model is shaded grey, and grey dots indicate the raw binary data of seedling occurrence (*n* = 67). (b) Graphical representation of the study site with dots as 0.5 × 0.5 m survey plots within the main sampling grid and triangles as 0.5 × 0.5 m survey plots within the superimposed grid. Positive seedling occurrences are indicated in red, and negative occurrences are indicated in black. The color‐scaled background is a kernel density at two meters of cadavers where darker colors represent higher cadaver density. (c) Young seedlings of the ericaceous shrub *Empetrum nigrum* establishing on disturbed substrate within our study site. Picture by Marie Davey

Shortly after the mass die‐off event (October 2016), a permanent, semi‐regular 10 × 10 m grid of 75 0.5 × 0.5 m survey plots was established, covering a 179 × 66 m area incorporating the mass die‐off and immediate surroundings (Appendix 1). The grid was established to monitor different aspects such as changes in vegetation, microbiota and animal interactions (e.g., by Steyaert et al., 2018 and Frank et al., 2020, though they used 1 × 1 m survey plots). Since it was difficult to predict the spatial scale of changes caused by the mass die‐off, the main grid was supplemented by a 10 × 10 m grid of 25 survey plots superimposed over the area of highest cadaver density (Appendix 1). In this paper, we use the data from the 2019 sampling campaign which incorporates 59 plots from the main grid and eight from the supplementary grid making 67 plots with a total area of 16.75 m^2^ (Figure 4b and Appendix 1; some plots from the original grids were not sampled in 2019 due to missing plot markers and logistical constraints). The grid overlays a gradient from undisturbed, intact vegetation cover through highly disturbed vegetation‐denuded patches around the cadavers, to persistent cadaver remains.

During the summer of 2019, we quantified seedling abundance of four ericaceous focal species: bilberry (*Vaccinium myrtillus*), bog bilberry (*V*. *uliginosum*), lingonberry (*V*. *vitis*‐*idaea*), and crowberry (*Empetrum nigrum*) within the 67 surveyed plots. To ensure constant sampling effort, each 0.5 × 0.5 m survey plot was divided into 16 subplots (12.5 × 12.5 cm), which were each systematically searched for 15 s by one of two trained researchers, totaling 4 min search time per survey plot. Seedlings were distinguished from small ramets originating from clonal propagation by the presence of seed‐leaves, while species identification was done using distinguishing features of the hypocotyl, seed‐leaves, epicotyl and first leaves (Muller, 1978). In each 0.5 × 0.5 m survey plot, the total cover of vascular plants, soil, persistent cadaver remains, stones, bryophytes and lichens were estimated to the nearest 5%.

Seedling age was not recorded, although different cohorts were observed among the seedlings counted in 2019. Many individuals had clearly germinated in 2019 appearing only with seed leaves and one or two first leaves—which also allowed for identification only to the genus level in some cases. However, some appeared to have germinated in the previous year and were elongated; amongst these, a few also had branching. Seedlings were not systematically inventoried in 2016–2018 but were expected to be remarked upon during vegetation surveys, given the importance of seedling establishment in the wider project at Vesle Saure. The presence of ericaceous seedlings at the site was indeed noted in the 2018 vegetation survey but not in 2016 or 2017.

### 2.3 Statistical analysis

We followed an information‐theoretic approach (Burnham & Anderson, 2002) to create and test candidate models as competing models for seedling establishment. We chose presence/absence of ericaceous seedlings at the survey plot (0.5 × 0.5 m) level as our response variable. Seedlings of ericaceous species were aggregated at the family level as there were too few observations for analysis of all but one species, *Empetrum nigrum*. Ericaceous seedling abundance was converted to a binary response variable (0 = seedlings not present, 1 = seedlings present) to avoid zero‐inflation and violation of model assumptions.

We included two sets of explanatory variables. The first set contained percentage cover of persistent cadaver remains within survey plots, distance to nearest cadaver from survey plot center and interpolated cadaver density reflecting the elevated seed rain observed closer to cadavers (Steyaert et al., 2018). Cadaver density was estimated using the kernel density function from ArcGIS 10.4 and across several search radii (bandwidths: 1–10 m in 1‐m increments). As a result, a smoothly curved surface was created over the study area based on cadaver density and the smoothness was determined by the number of cadavers within a neighborhood and the size of the neighborhood (bandwidth used). To determine the optimal spatial scale for seedling establishment, we fitted each bandwidth in one‐predictor generalized linear models (GLMs) with a Bernoulli distribution and seedling presence/absence as the response variable. We used Akaike Information Criterion corrected for small sample sizes (AICc) for model selection (Burnham & Anderson, 2002) and the “best” model with the lowest AICc value (search radius 2 m; Appendix 2) supplied the model term for further analysis. The second set of explanatory variables consisted of percentage cover of vascular plants, lichens, bryophytes and bare soil as measures of changed microsite conditions (e.g., competition) within the survey plots which have undergone disturbance. The rationale for including individual explanatory variables in each set is further elaborated in Table 1.

**TABLE 1:** The set of 14 *a priori* candidate models to explain seedling establishment (presence/absence) of berry‐producing ericaceous shrub species

Model	Terms	Rationale	Category
1	Cadaver	Presence of persistent cadaver material facilitates continued elevated seed rain (i.e., scat deposition)	Cadaver location
2	CadDen	High cadaver density facilitates CDI's (i.e., RWOs) with elevated seed rain	
3	CadDist	Distance to nearest cadaver affects likelihood of CDI formation and scat deposition rate	
4	CadDen + CadDist	Cadaver density and proximity facilitates CDI’s with elevated seed rain	
5	Cadaver + CadDist	Cadaver proximity facilitates CDI formation while persistent cadaver material within in plots provides continued seed rain	
6	Plant	Seedling establishment is reduced by competition from other vascular plants	Microsite conditions
7	Bryophyte	Bryophytes may either function as competition or nursery plants to establishing seedlings	
8	Soil	Areas with less competition will positively influence seedling establishment	
9	Plant + Lichen + Bryophyte	Competition negatively affects seedling establishment	
10	CadDen + Soil	Cadaver density facilitates CDI and elevated seed rain while high soil cover (i.e., low competition) positively affects seedling establishment	Combination
11	CadDist + Plant	Long distance to cadavers and competition from vascular plants negatively affects seedling establishment	
12	CadDen * Soil	Cadaver density facilitates CDI and enhanced seed rain but highly disturbed plots (i.e., high soil cover) are not ready for revegetation	
13	CadDen + CadDist + Soil	Cadaver density and proximity facilitates CDI and high seed rain while high soil cover positively affects seedling establishment	
14	Null	Intercept only	

Note

The candidate model set encompassed three subsets: (I) models based on cadaver location, reflecting the elevated seed rain (Steyaert et al., 2018) observed closer to cadavers, II) models based on plant and soil cover, reflecting the reduced competition in the plots which have undergone disturbance, and (III) cadaver location and microsite condition combination models. Cover of cadaver, plant (i.e., vascular), lichen and bryophytes is expressed as percentage cover within survey plots. CadDen is cadaver kernel density (search radius = 2 m) and CadDist is distance to nearest cadaver from survey plot. Candidate models were fitted using generalized linear models (GLMs) with a Bernoulli distribution.

We assessed collinearity between explanatory variables prior to building candidate models using variance inflation factors (VIF) and Pearson's *r* correlation coefficient (Zuur et al., 2010). Collinear variables (VIF < 2 and Pearson's *r* < .6) were not included within the same candidate models.

Using the variables described above, a set of 14 biologically meaningful *a priori* candidate models were developed including a null model (intercept only) to explain seedling establishment. The candidate model set encompassed three subsets: (I) models based on cadaver location only (II) models based on microsite conditions only, (III) cadaver location and microsite condition combination models (Table 1). We allowed for interaction terms where such an interaction could plausibly have an effect on seedling establishment. Due to the relatively small sample size, we restricted our models to combinations of ≤3 explanatory variables and fitted them using GLMs with a Bernoulli distribution.

We ranked the competing models according to AICc value and considered models within two AICc units of the top ranked model (∆AICc ≤ 2.0) to have substantial support from the data and be part of the top model set (Burnham & Anderson, 2002). Nested models, that is, more complex versions of higher ranked models may produce “pretender variables” which have no relationship with the response variable and do not improve model fit (Burnham & Anderson, 2002). To avoid the inclusion of such uninformative variables (CI overlapping zero) within the top model set, nested models were not considered as competing models for seedling establishment (Arnold, 2010; Leroux, 2019).

We examined model residuals to ensure that assumptions were met and that effects were adequately accounted for by the model. Following Dormann et al. (2007) we assessed spatial autocorrelation of seedling presence/absence with a Moran's I test on model residuals using the R package “spdep” (Bivand & Wong, 2018) and detected no spatial dependency (*p*‐value = .62). All statistical analyses were performed in R 4.0.2 (R Core Team, 2020).

## 3 RESULTS

In total, we registered 43 ericaceous seedlings distributed over 12 of the 67 surveyed plots (range: 1–10 seedlings per survey plot). *E*. *nigrum* was most abundant (*n* = 22), followed by *V*. *vitis*‐*idaea* (*n* = 11), *V*. *uliginosum* (*n* = 7) and *V*. *myrtillus* (*n* = 1). Because of their early juvenile state, two seedlings could not be identified beyond genus level (*Vaccinium* spp.).

The best model for predicting seedling presence contained the single explanatory variable cadaver density (Table 2). The probability of detecting ericaceous seedlings was positively associated with cadaver density (*β* = 7.224, SE = 2.296, *p*‐value <.01; Figure 4a). Cadaver density was included in all four models within the top model set (∆AICc ≤ 2.0) and of the next three models, two contained soil cover and one contained distance to nearest cadaver (Table 2). However, in none of these models either of the parameters or their interaction had a significant effect on seedling establishment.

**TABLE 2:** The set of 14 *a priori* candidate models ranked according to decreasing AICc

Model	Model terms	df	AICc	ΔAICc	*w_i_*
2	*CadDen*	2	51.708	0	0.308
12	*Soil* × CadDen	4	52.363	0.655	0.222
10	*Soil* + *CadDen*	3	52.923	1.215	0.168
2	*CadDen* + *CadDist*	3	52.927	1.219	0.168
13	*Soil* + *CadDen* + *CadDist*	4	54.566	2.858	0.074
3	*CadDist*	2	57.454	5.746	0.017
11	*Plant* + *CadDist*	3	57.826	6.118	0.014
5	*CadDist* + *Cadaver*	3	58.122	6.414	0.012
1	*Cadaver*	2	59.810	8.101	0.005
6	*Plant*	2	60.468	8.760	0.004
8	*Soil*	2	60.789	9.081	0.003
9	*Bryophyte* + *Plant* + *Lichen*	4	61.677	9.969	0.002
7	*Bryophyte*	2	63.232	11.524	0.001
14	*Null*	1	65.046	13.338	0.000

Note

Note that model 12, 10 and 2 (ΔAICc < 2) are not considered to be competing models as they are simply more complex versions of the top‐ranked model.

Abbreviations: AICc, Akaike Information Criterion corrected for small sample size; df, degrees of freedom; *w*, model weight; ΔAICc, AICc difference values compared to the model with the lowest AICc value.

## 4 DISCUSSION

Cadaver decomposition islands created by ungulate cadavers provide microsites that are particularly suitable for ericaceous seedling establishment, as demonstrated by the findings within our study. This extends the findings of Steyaert et al. (2018), in which cadavers were identified as endpoints for directed endozoochorous dispersal by scavenging omnivores. Further, by encompassing a gradient from undisturbed, intact vegetation cover through highly disturbed denuded patches around the cadavers, to persistent cadaver remains, we show that favorable microsites characteristics alone are inadequate for seedling establishment. The amount of bare soil and vegetation cover—which represent important microhabitat conditions—were poor predictors of seedling establishments. Similarly, disturbed survey plots with low vegetation cover (i.e., low competition) which were adjacent to mature stands of ericaceous shrubs in which we observed ripe berries (and so seed rain is expected to be relatively high due to berries falling; Graae et al., 2011) did not show seedling establishment unless they were also near a cadaver. Alternatively, this absence could be due to the short distance to conspecific adults were seeds and seedlings can suffer higher mortality from the activity of specialized natural enemies such as seed predators (Comita et al., 2014). However, ericaceous species such as *V*. *myrtillus* exhibit masting (Selås, 2000) and thus can be expected to experience weak density–distance regulation of seed and seedling mortality (Bagchi et al., 2011), possibly due to seed predator satiation (Janzen, 1970). The lack of observed seedling establishment under natural or experimental disturbance (e.g., Eriksson & Fröborg, 1996; Hautala et al., 2001; Hester et al., 1991; Manninen & Tolvanen, 2017) may be a case of looking for a needle in the wrong haystack: our results imply that seedling distribution may be related as much to the activity of endozoochorous dispersers (i.e., getting to the right place) as it is to habitat disturbance (Garcia‐Cervigon et al., 2018; Schupp et al., 2010).

The somewhat, but not entirely clustered seedling distribution in close proximity to cadavers precludes several alternative explanations for the occurrence of these seedlings. For example, Bråthen et al. (2007) found approximately five viable seeds of ericaceous berry‐producing species per liter of reindeer feces in northern Norway. If the source of the seedlings were the gut contents of the cadavers themselves, the seedlings would occur only directly upon the cadavers rather than around them. If frugivorous or granivorous species such as lemmings (*Lemmus lemmus*) or ptarmigan (*Lagopus muta*) were depositing seeds without acting as cadaver scavengers (i.e., random seed dispersal not directed at cadavers), seedling occurrence would not be clustered around the cadavers—quite possibly the opposite. Frank et al. (2020) found that the cadavers created a landscape of fear where rodents avoided the CDI due the presence of facultative scavenger species which might predate the rodents. Finally, the seedbanks of ericaceous seed species are mostly transient and contain few seeds (Thompson et al., 1997; Welch et al., 2000). Although several studies report more persistent seedbanks (Thompson et al., 1997), if seedlings had originated from the seedbank we would expect a more even distribution within the CDI. Similarly, stronger seed limitation than microsite limitation has been described for recruitment of berry‐producing ericaceous species in forest and alpine ecosystems (Manninen & Tolvanen, 2017): seedling recruitment into favorable microsites such as disturbances is significantly enhanced by seed addition (e.gEriksson & Fröborg, 1996; Hautala et al., 2001; Manninen & Tolvanen, 2017; Welch et al., 2000). For example, in Hautala et al.’s (2001) unsown plots they recorded zero to four seeds m^−2^ depending on disturbance regimes, whereas sown plots had seedling densities ranging from 1.2 to 56.4 seedlings m^−2^. Our survey plot with the most seedlings had ten seedlings m^−2^.

For those berry‐producing ericaceous species which present a reproductive paradox, the apparent conundrum lies in the absence of a seedbank. Such species are dependent on elevated seed rain at suitable microsites for germination and subsequent establishment (Eriksson & Fröborg, 1996; Manninen & Tolvanen, 2017). We show that vertebrate facultative scavengers may be a critical component of the regeneration niche of ericaceous species: plants may rely upon directed endozoochorous seed rain arriving at suitable microsites, in this case a relatively short‐lived RWO provided by a CDI (Figure 1).

More recent studies on several *Vaccinium* and *E*. *nigrum* populations have documented higher genetic diversity than expected for clonal populations (Albert et al., 2004, 2005; Bienau et al., 2016; Persson & Gustavsson, 2001). This indicates that they establish from seed to a greater extent than previously thought including from outside their immediate genetic neighborhood (Jordano, 2017). For long‐lived clonal plant populations even occasional seedling recruitment can be sufficient for maintaining genetic diversity in (De Witte et al., 2012; Watkinson & Powell, 1993).

The large home ranges of medium‐ to large‐sized scavenger species, such as those observed within our study site, combined with their long gut retention times (Cunze et al., 2013), suggests the potential for long‐distance dispersal *senso stricto*, that is dispersal outside the range and genetic neighborhood of the parental population (Jordano, 2017). Despite their apparent rarity, such “true” long distance dispersal events are known to play a major role in large‐scale dynamics of plant populations as they promote gene flow between populations, colonization of unoccupied habitat and range expansion (Cain et al., 2000; Nathan et al., 2008). Even medium‐sized scavengers considerably outperform co‐occurring smaller seed dispersal vectors such as small and medium‐sized passerine birds in providing a long‐distance seed dispersal service for fruit‐producing species (Jordano, 2017; Jordano et al., 2007). The combination of scavengers’ potential effectiveness as long‐distance dispersal vectors and the directed nature of the seed dispersal shadow (Steyaert et al., 2018) indicate their possible efficacy and importance as dispersers for plant species exploiting ephemeral RWOs.

We took advantage of a large mass die‐off event in a relatively undisturbed mountain area to demonstrate that directed dispersal toward CDIs facilitates seedling establishment. Cadavers at the study site persisted over several years and continued to attract scavenging omnivores during the ericaceous berry season (Frank et al., 2020)—a crucial point for this mechanism to work. Although mass die‐off events have been consistently reported over the past decades (Fey et al., 2015), large numbers of terrestrial herbivores also die from natural causes (e.g., predation, starvation, or disease) leaving single cadavers in the landscape (Barton et al., 2019; Moleón et al., 2019). Such single cadavers may be regularly provided throughout the year by for example large predators that only partly consume prey (Wilmers et al., 2003). However, at northern latitudes, maximum cadaver availability is in late winter when ungulate mortality is high due to starvation and thermal stress, creating temporally aggregated pulses of available carrion (Pereira et al., 2014). Factors such as the spatial and temporal distribution of cadavers, cadaver size, and environmental variables affect both the development and size of CDIs (Towne, 2000). Moreover, the assembly of scavenger guilds (i.e., disperser species) utilizing cadavers (Selva et al., 2005; Turner et al., 2017) and the interactions between a cadaver, scavengers and surrounding habitat may have a considerable impact on whether CDIs, directed dispersal, or seedlings occur. For example, a cadaver deposited in winter might be either completely consumed or scattered over the landscape by scavengers within a relatively short time (Towne, 2000; Turner et al., 2017) and thus not persist until berry season or form a CDI. In alpine and arctic regions, however, cadavers may persist longer within the landscape, as we observed at Vesle Saure where cadaver material was still in place in 2019. Hence, it remains unclear how this mechanism works with single carcass events, across different ecosystems, and with different scavenger guilds.

Anthropogenic pressures on ecosystems continue to rise (Dirzo et al., 2014) and humans are likely disrupting key ecosystem functions, for example those provided by vertebrate scavengers (Mateo‐Tomás et al., 2017; Sebastián‐González et al., 2019, 2020). This could be particularly detrimental to long‐term plant population resilience when, as we show, some plant species may benefit from the endozoochorous dispersal service provided by facultative scavengers to facilitate recruitment and so completion of their life cycle (Traveset et al., 2012). Our study area had a relatively intact scavenger guild, but anthropogenic pressures are significantly affecting scavenger richness and abundance on a global scale (Sebastián‐González et al., 2019, 2020). Humans can affect scavenger species directly through persecution (Swenson et al., 1995) but also indirectly through cadaver removal (Margalida et al., 2010), habitat fragmentation and destruction (Sebastián‐González et al., 2019). Furthermore, a reduction in cadaver availability would also decrease the frequency of CDIs, that is, reduce microsite availability. Seed dispersal shadows and long‐distance dispersal events strongly depend on the relative contributions of different scavenging disperser species (González‐Varo et al., 2013). The loss of a vector providing effective directed dispersal could decrease genetic diversity at the population level, potentially resulting in a marked reduction in population fitness (Wenny, 2001).

Our study provides a novel understanding of sexual reproduction in species with cryptic generative reproduction and to our knowledge, we are the first to observe this mechanism in action. This is proof‐of‐concept and demonstrates how directed dispersal toward CDIs mediates a higher probability of seedling establishment, giving support that directed seed dispersal by facultative scavengers toward CDIs provides a pathway to successful sexual reproduction in berry‐producing ericaceous shrubs. However, the relative importance of this dispersal mechanism to population persistence in the long term and how humans influence it is not clear and may only be elucidated through studies on the genetic structure of populations of these plants.

## CONFLICT OF INTEREST

The authors have no competing interests.

## AUTHOR CONTRIBUTIONS

**Mie Prik Arnberg:** Formal analysis (lead); investigation (equal); methodology (equal); visualization (lead); writing – original draft (lead); writing – review and editing (equal). **Shane C. Frank:** Conceptualization (equal); formal analysis (supporting); investigation (equal); methodology (equal); writing – review and editing (equal). **Rakel Blaalid:** Investigation (equal); methodology (equal); writing – original draft (supporting); writing – review and editing (equal). **Marie Louise Davey:** Investigation (equal); methodology (equal); writing – review and editing (equal). **Amy Elizabeth Eycott:** Writing – original draft (supporting); writing – review and editing (equal). **Sam M. J. G. Steyaert:** Conceptualization (equal); investigation (equal); methodology (equal); visualization (supporting); writing – review and editing (equal).

## Data Availability

The data used in this study is deposited in the Dryad Digital Repository: https://doi.org/10.5061/dryad.fn2z34ttz.

## APPENDIX 1

Graphical representation of the sampling grid setup at the study site. Dots represent the 75 survey plots (0.5 × 0.5 m) in the semiregular 10 × 10 m main grid. Triangles are the supplementary 25 survey plots superimposed in a 10 × 10 m grid over the area of highest cadaver density. Both grids were established shortly after the mortality event in October 2016. The grey dotted line indicates the 2019 study area, in which green colored survey plots were sampled, while black survey plots were not due to missing plot markers or logistical constraints. Brown dots are cadaver positions (*n* = 323).

## APPENDIX 2

Model selection results from single predictor models (GLMs) relating seedling establishment (yes/no) to the different spatial scales for estimating cadaver kernel density (search radii 1–10 m in 1 m increments). The search radius for estimating the cadaver kernel density follows the “Cadaver density” model term in parentheses. AICc, Akaike Information Criterion corrected for small sample size; ΔAICc, AICc difference values compared to the model with the lowest AICc value; *w*, model weight.

**Table:**

Model term	df	AICc	ΔAICc	*w_i_*
Cadaver density (2 m)	2	51.708	0	0.370
Cadaver density (3 m)	2	52.649	0.941	0.231
Cadaver density (4 m)	2	54.263	2.554	0.103
Cadaver density (5 m)	2	55.013	3.304	0.071
Cadaver density (6 m)	2	55.578	3.870	0.053
Cadaver density (7 m)	2	55.826	4.117	0.047
Cadaver density (8 m)	2	55.992	4.284	0.043
Cadaver density (9 m)	2	56.199	4.491	0.039
Cadaver density (10 m)	2	56.382	4.673	0.036
Cadaver density (1 m)	2	60.290	8.581	0.005
